# Study protocol for pragmatic trials of Internet-delivered guided and unguided cognitive behavior therapy for treating depression and anxiety in university students of two Latin American countries: the *Yo Puedo Sentirme Bien* study

**DOI:** 10.1186/s13063-022-06255-3

**Published:** 2022-06-02

**Authors:** Corina Benjet, Ronald C. Kessler, Alan E. Kazdin, Pim Cuijpers, Yesica Albor, Nayib Carrasco Tapias, Carlos C. Contreras-Ibáñez, Ma Socorro Durán González, Sarah M. Gildea, Noé González, José Benjamín Guerrero López, Alex Luedtke, Maria Elena Medina-Mora, Jorge Palacios, Derek Richards, Alicia Salamanca-Sanabria, Nancy A. Sampson

**Affiliations:** 1 Center for Global Mental Health, National Institute of Psychiatry Ramón de la Fuente Muñiz, Mexico City, Mexico; 2 Department of Health care Policy, Harvard Medical School, Boston, MA USA; 3 Yale University, New Haven, CT USA; 4 Department of Clinical, Neuro and Developmental Psychology, Amsterdam Public Health Research Institute, Vrije Universiteit Amsterdam, Amsterdam, Netherlands; 5 Center for Global Mental Health, National Institute of Psychiatry Ramón de la Fuente Muñiz and School of Psychology, UNAM, Mexico City, Mexico; 6 Universidad Cooperativa de Colombia, Medellin, Colombia; 7 Universidad Autónoma Metropolitana, Mexico City, Mexico; 8 Universidad De La Salle Bajío, León, GT Mexico; 9 Departamento de Psiquiatría y Salud Mental, Universidad Nacional Autónoma de México, Mexico City, Mexico; 10 Department of Statistics, University of Washington, Seattle, WA USA; 11 SilverCloud Health, Dublin, Ireland; 12 E-mental Health Group, School of Psychology, University of Dublin, Trinity College Dublin, Dublin, Ireland; 13 Future Health Technologies Programme, Campus for Research Excellence and Technological Enterprise, Singapore-ETH Centre, Singapore, Singapore

**Keywords:** Depression, Anxiety, iCBT, Latin America, College students, Precision treatment algorithm

## Abstract

**Background:**

Major depressive disorder (MDD) and generalized anxiety disorder (GAD) are highly prevalent among university students and predict impaired college performance and later life role functioning. Yet most students do not receive treatment, especially in low-middle-income countries (LMICs). We aim to evaluate the effects of expanding treatment using scalable and inexpensive Internet-delivered transdiagnostic cognitive behavioral therapy (iCBT) among college students with symptoms of MDD and/or GAD in two LMICs in Latin America (Colombia and Mexico) and to investigate the feasibility of creating a precision treatment rule (PTR) to predict for whom iCBT is most effective.

**Methods:**

We will first carry out a multi-site randomized pragmatic clinical trial (*N* = 1500) of students seeking treatment at student mental health clinics in participating universities or responding to an email offering services. Students on wait lists for clinic services will be randomized to unguided iCBT (33%), guided iCBT (33%), and treatment as usual (TAU) (33%). iCBT will be provided immediately whereas TAU will be whenever a clinic appointment is available. Short-term aggregate effects will be assessed at 90 days and longer-term effects 12 months after randomization. We will use ensemble machine learning to predict heterogeneity of treatment effects of unguided versus guided iCBT versus TAU and develop a precision treatment rule (PTR) to optimize individual student outcome. We will then conduct a second and third trial with separate samples (*n* = 500 per arm), but with unequal allocation across two arms: 25% will be assigned to the treatment determined to yield optimal outcomes based on the PTR developed in the first trial (PTR for optimal short-term outcomes for Trial 2 and 12-month outcomes for Trial 3), whereas the remaining 75% will be assigned with equal allocation across all three treatment arms.

**Discussion:**

By collecting comprehensive baseline characteristics to evaluate heterogeneity of treatment effects, we will provide valuable and innovative information to optimize treatment effects and guide university mental health treatment planning. Such an effort could have enormous public-health implications for the region by increasing the reach of treatment, decreasing unmet need and clinic wait times, and serving as a model of evidence-based intervention planning and implementation.

**Trial status:**

IRB Approval of Protocol Version 1.0; June 3, 2020. Recruitment began on March 1, 2021. Recruitment is tentatively scheduled to be completed on May 30, 2024.

**Trial registration:**

ClinicalTrials.govNCT04780542. First submission date: February 28, 2021.

## Administrative information

Note: the numbers in curly brackets in this protocol refer to SPIRIT checklist item numbers. The order of the items has been modified to group similar items (see http://www.equator-network.org/reporting-guidelines/spirit-2013-statement-defining-standard-protocol-items-for-clinical-trials/).

**Table:** 

Title {1}	Study protocol for pragmatic trials of internet-delivered guided and unguided cognitive behavior therapy for treating depression and anxiety in university students of two Latin American countries: The *Yo Puedo Sentirme Bien* Study.
Trial registration {2a and 2b}.	ClinicalTrials.gov ID: NCT04780542
Protocol version {3}	First submission date: February 28, 2021
Funding {4}	This trial is funded by US the National Institute of Mental Health and Fogarty International Center R01MH120648
Author details {5a}	Corina Benjet, PhD; Center for Global Mental Health, National Institute of Psychiatry Ramón de la Fuente Muñiz, Mexico City, Mexico Ronald C. Kessler, PhD; Department of Health Care Policy, Harvard Medical School, Boston, MA Alan E. Kazdin, PhD; Yale University, New Haven, CT, USA Pim Cuijpers, PhD; Department of Clinical, Neuro and Developmental Psychology, Amsterdam Public Health Research Institute, Vrije Universiteit Amsterdam, Netherlands Yesica Albor, PhD; National Institute of Psychiatry Ramón de la Fuente Muñiz, Mexico City, Mexico Nayib Carrasco Tapia, PhD; Universidad Cooperativa de Colombia, Medellin, Colombia Carlos C. Contreras-Ibáñez, PhD; Universidad Autónoma Metropolitana, Mexico City, Mexico Ma. Socorro Durán González, MA; Universidad De La Salle Bajío Sarah M. Gildea, BS; Department of Healthcare Policy, Harvard Medical School, Boston, MA Noé González, MA; Instituto Nacional de Psiquiatría Ramón de la Fuente Muñiz, Mexico City, Mexico José Benjamín Guerrero López, Departamento de Psiquiatría y Salud Mental, Universidad Nacional Autónoma de México, Mexico City, Mexico Alex Luedtke, PhD; Department of Statistics, University of Washington, Seattle, WA, USA Maria Elena Medina-Mora, PhD; Center for Global Mental Health, National Institute of Psychiatry Ramón de la Fuente Muñiz and School of Psychology, UNAM, Mexico City, Mexico Jorge Palacios, PhD; SilverCloud Health, Dublin, Ireland and E-mental Health Group, School of Psychology, University of Dublin, Trinity College Dublin, Ireland Derek Richards, PhD; SilverCloud Health, Dublin, Ireland and E-mental Health Group, School of Psychology, University of Dublin, Trinity College Dublin, Ireland Alicia Salamanca-Sanabria, PhD; Future Health Technologies Programme, Campus for Research Excellence and Technological Enterprise, Singapore-ETH Centre, Singapore, Singapore Nancy A. Sampson; Department of Healthcare Policy, Harvard Medical School, Boston, MA Ronald C. Kessler, PhD; Department of Health care Policy, Harvard Medical School, Boston, MA
Name and contact information for the trial sponsor {5b}	National Institute of Mental Health and Fogarty International Center, Pim Brouwers Program Officer, 6001 Executive Blvd, Rockville, MD, 20892 USA
Role of sponsor {5c}	The US NIMH and FIC fund this trial. The funders are not involved in the study design, collection, management, analysis, or interpretation of data, writing of the report, or the decision to submit the report for publication.

## Introduction

### Background and rationale {6a}

The World Mental Health (WMH) community surveys [1] across 21 countries (including Colombia and Mexico) documented high prevalence, unmet need for treatment, and associated college attrition due to common mental disorders (CMDs) among university students. As a result, WMH launched the WMH-International College Student (ICS) initiative to implement inexpensive Internet-based needs assessment surveys to help evaluate the treatment gap and barriers to obtaining treatment of CMDs [2]. WMH-ICS surveys have so far been carried out in 15 mostly high-income countries and are planned for other countries. These surveys confirm the high prevalence [3], impairment [4], and unmet need for treatment [5] of CMDs among college students, especially in low- and middle-income countries (LMICs), where only 10–20% of students with CMDs receive any treatment [6–8]. Importantly, the proportion of young people in LMICs who attend college is small and these students have particular needs [9], as the rapid growth of tertiary education in these countries means that many are first-generation college students [10, 11] who have low socialization into the challenges of college life, high financial stress, and in many cases high pressures to succeed to help support their extended families [12, 13]. These special challenges create high levels of emotional problems [14, 15] that often interfere with academic success [16, 17]. However, institutions of higher education in LMICs, despite the important public investment in these students’ higher education, are not well-equipped to deal with these problems due to their low staff-to-student ratios and low resources to manage student CMDs [11, 18]. We seek to evaluate and optimize the effectiveness of low-cost self-guided and supporter-guided Internet-delivered transdiagnostic cognitive behavioral therapy (iCBT) to provide a feasible way to address the enormous unmet need for treatment in this critical segment of the LMIC population [9].

Internet-delivered interventions may be particularly well suited for college students as they address many barriers to treatment in this population, such as scheduling and transportation, cost, and stigma while fostering a sense of control. Whereas Internet-delivered interventions are becoming increasingly common in the USA and Europe [19], they remain under-utilized in LMICs [20, 21] in general and in Latin America in particular [22]. Importantly, existing research on Internet interventions in LMICs shows that such interventions are feasible and at least as effective as they are in high-income countries [20–22]. Literacy and Internet access, which limit Internet interventions in the general population of many LMICs, are not obstacles among college students, most of whom have email addresses (often provided by their universities), smart phones, and access to computers with Internet access.

Five project innovations are noteworthy. First, few controlled trials of Internet-based interventions have been implemented for CMDs in LMICs [20–22]. Our study is innovative in using iCBT to treat LMIC university students. iCBT has been widely used among college students in high-income countries with multiple meta-analyses attesting to its effectiveness [23, 24]. A recent meta-analysis of the effectiveness of digital interventions for mental health in LMICs [21] found that only one country in Latin-America, Colombia, implemented an iCBT study among university students. That study’s outcomes support the potential of using iCBT for university students in Latin American countries [25].

Second, the iCBT program we plan to use, *SilverCloud* (see below), is a transdiagnostic program [26, 27] focused on treatment of mood and anxiety disorders, either with or without a number of other comorbid disorders (such as eating disorders, insomnia, or substance use disorders). This is important because WMH-ICS data show that comorbidity is highly prevalent among LMIC college students [3] and strongly predicts role impairment [4]. Evaluation of transdiagnostic interventions is a high-priority National Institute of Mental Health research area (NIMH Strategic Objective 3.1) [28].

Third, while most trials evaluate aggregate effects of treatment, growing evidence suggests heterogeneity of treatment effects (HTE) such that different types of people benefit from different types of treatment [29]. Machine learning can use a large number of baseline characteristics to estimate a prediction model for each treatment arm, use counter-factual logic to assign predicted outcome scores to each student for each treatment, and examine individual difference scores to decide which treatment is best for each student. This information could be very useful for deciding what type of treatment to offer different types of students. Therefore, following an initial pragmatic clinical trial from which we will develop a precision treatment algorithm based on machine learning, we will attempt to evaluate the implications of using that rule in a second and third trial carried out in subsequent years to evaluate the extent to which the PTR would improve on randomization.

Fourth, we will investigate barriers to seeking treatment by carrying out a needs assessment survey in parallel with the intervention and examining patterns and correlates of barriers to seeking treatment. Students who are identified in the needs assessment survey as meeting the same depression and anxiety criteria as specified for the main trial but who do not seek treatment at the clinic will be offered the opportunity to participate in the trial. Depending on information obtained about barriers to treatment, the outreach efforts to recruit these students might change over the course of the trial. If so, information about variation in these efforts will be included in developing the PTR. With this ancillary trial, we will evaluate the extent to which iCBT, by providing a confidential and convenient way of obtaining treatment, can increase the proportion of students with CMDs who seek treatment. This is important because the proportion of students with CMDs who seek treatment currently is quite low. Several other interventions like this could easily be envisioned, such as campus-wide interventions to increase awareness of mental health clinics and reduce stigma [30]. However, universities are understandably reluctant to implement such interventions and it would be unethical to do so prior to having access to and evidence for the effectiveness of scalable interventions, like guided or un-guided iCBT. Based on this practical and ethical reality, we believe that a good first step in investigating opportunities for expanding treatment is the one proposed here and that the infrastructure created by this project can subsequently be used to facilitate implementation and evaluation of other outreach interventions aimed at addressing the problem of unmet need for treatment.

Fifth, we will use an innovative ensemble machine learning precision treatment modeling method [31] to develop the PTR. In addition to using an innovative modeling approach, we will include a much more extensive set of prescriptive predictors (*i.e., predictors of individual differences in the effects of CBT compared either to no treatment or other types of treatment* [32]) in the baseline assessment than in prior efforts to develop a PTR for CMD treatment. Prior studies typically examined only a handful of prescriptive predictors [33]. The prescriptive predictors we use will include all those found consistently in prior studies [32].

Given the current situation of the global coronavirus disease 2019 (COVID-19) pandemic, there is a growing need and growing acceptance of interventions delivered at a distance. This trial is timely with the potential to provide valuable knowledge and tools to meet these challenges. Moreover, the implications for treatment delivery will extend well beyond the circumstances (e.g., confinement) caused by the current pandemic.

## Objectives {7}

The overall goals of this study are to evaluate the effects of expanding treatment of CMDs among college students with symptoms of major depressive disorder (MDD) and/or generalized anxiety disorder (GAD) in two LMICs in Latin America (Colombia and Mexico) and to investigate the extent to which treatment outcomes could be improved by creating precision treatment algorithms to predict which intervention would be best for which students. The intervention will be a scalable and inexpensive intervention: iCBT. We have four specific objectives.

Objective 1: To carry out a year 1 pragmatic clinical trial with students in participating universities in Colombia and Mexico. Students will be recruited both from student mental health clinic waiting lists and from outreach emails sent to probability samples of other students. The student clinics have long waiting lists for all but the most urgent cases. Students on wait lists will be offered a two-thirds chance to receive iCBT immediately while staying on the list. The same offer will be made to students from the general student body recruited from emails. Interested students will be required to complete a baseline self-report assessment and, if eligible, to be randomized with equal allocation either to unguided iCBT (33%), guided iCBT (33%), or treatment as usual (TAU; that is, they will remain on the clinic waiting list until their appointment). Short-term aggregate intervention effects among students randomized across arms will be assessed 90 days after randomization and longer-term effects 12 months after randomization.

Objective 2: To use ensemble machine learning methods to predict heterogeneity of treatment effects of unguided iCBT versus guided iCBT versus TAU and develop a PTR to predict which students will respond best to which intervention. We will build a PTR for 90-day remission and a separate PTR for 12-month remission based on the year 1 pragmatic trial.

Objective 3: To carry out two additional trials in years 2 and 3 that build on the results of the first trial. Recruitment will be the same as in the first trial but randomization will be different in that a random 25% of participants will be randomized to the treatment estimated by the PTR for short-term response (the year 2 trial) or 12-month remission (the year 3 trial) based on the year 1 trial and the remaining 75% will be randomized with equal allocation across all 3 treatments.

Objective 4: The technology for delivering iCBT, developing and updating PTRs, and carrying out Internet-based mental health needs assessment surveys will be disseminated to colleges and universities in LMICs throughout Latin America. This dissemination will occur through conferences, seminars, and responding to requests for consultation.

## Trial design {8}

First, we will conduct a three-arm single-blind parallel assignment individually randomized equal allocation controlled pragmatic trial on *n* = 1500 students. It will compare aggregate effects of unguided iCBT, guided iCBT, and TAU at 90 days and 12 months. Consistent with previous research, we hypothesize that guided iCBT will have superior aggregate treatment effects to unguided iCBT, but as this will be an initial study that combines novel treatments, methods of treatment delivery, and new ways of matching treatments to students, we were not able to draw on prior theory or research to hypothesize whether the aggregate treatment effects of iCBT will be superior to those of TAU. However, we hypothesize that comparative effectiveness across the three arms will vary significantly by student baseline socio-demographic, environmental, and clinical characteristics,

This initial trial will be followed by two additional trials using the same recruitment methods and same interventions but differing in allocation rules. In the second trial, 25% of n = 1000 students will be individually assigned to the treatment estimated by the PTR in the first trial to yield an optimal short-term response for the student, with the remaining 75% randomized with equal allocation across all 3 treatments. In the third trial, 25% of n = 1000 students will be individually assigned to the treatment estimated by the PTR in the first trial to yield an optimal 12-month probability of remission for the student, with the remaining 75% randomized with equal allocation across all 3 treatments. We hypothesize that students assigned to the treatment recommended by the PTRs will have significantly better outcomes than those randomized across types of treatment. These additional trials will provide a direct evaluation of the value of the PTRs.

## Methods: participants, interventions, and outcomes

### Study setting {9}

Participants will be undergraduate students at public and private universities in Colombia and Mexico, beginning with Universidad Cooperativa de Colombia, la Red de Universidades la Salle, Universidad Autónoma Metropolitana, and Universidad Nacional Autónoma de México. However, it is expected that several more universities will be added. Students will be recruited both from the university student mental health clinics and from the general population of non-help seeking students.

### Eligibility criteria {10}

All undergraduate students at the participating universities that are at least 18 years of age and who screen positive for elevated depressive and/or anxious symptomatology will be eligible to participate irrespective of whether they also present symptomatology of other CMDs like adult ADHD, substance use disorders, and PTSD. Exclusion criteria include any one of the following: (1) screening positive for possible bipolar disorder, (2) report having received a diagnosis of psychosis, or (3) are considered to have serious suicide risk based on self-report of suicide ideation with current intent.

### Who will solicit informed consent? {26a}

Consent procedures were approved by the IRB of the Instituto Nacional de Psiquiatría Ramón de la Fuente Muñiz, the coordinating center. For students recruited from the university mental health clinics, the intake person at the clinic will identify potential study participants when the participants ask for an appointment and will describe the study and answer any questions. Subsequently, interested students will be sent an email invitation and link to the study. Students recruited from the representative samples generated from the list of enrolled students or from those who responded to announcements on the university social media platforms will receive an email that describes the study with an attached study brochure and will be offered to receive a phone call if they would like to ask more questions or receive an explanation from the study team. All students will be able to provide consent online.

### Additional consent provisions for collection and use of participant data and biological specimens in ancillary studies {26b}

If we decide to do an ancillary study in the future, we will seek prior consent of participants to be re-contacted and re-consent for the ancillary study following IRB approval; we do not collect any biological samples.

### Interventions

#### Explanation for the choice of comparators {6b}

CBT is recommended as a first-line treatment for depression and anxiety [34]. iCBT is becoming increasingly common in high-income countries [35] for many reasons including insufficient psychotherapists trained in CBT, the advantages in terms of accessibility (convenience in schedule and travel), empowerment (increased sense of control and psychological ownership), and reduction of barriers (reduced cost, logistical barriers, wait times, and discretion for those who are embarrassed to seek help). Several iCBT programs, both guided and unguided with and without modules to address comorbid disorders, have been developed, implemented, and evaluated (for reviews, see [36–38]). The online platform chosen here, developed by SilverCloud Health, is widely used both commercially (e.g., [39]) and in research (e.g., [27, 40, 41]). It encompasses a wide range of programs, treating an array of conditions from insomnia and stress to comorbid depression and anxiety. The specific program we will use (called *Space from Depression and Anxiety* in the English version) was modified specifically for Latin American college students for this study. This modification was based on a prior cultural adaptation developed for Colombia [42] for a depression-only version translated as *Yo Puedo Sentirme Bien* [“I can feel good” in English]. This name was evaluated in focus groups to be more culturally appropriate than the direct translation. Cross-cultural principles were considered, in which focus group discussions were conducted with Mexican and Colombian students to evaluate the relevance of the personal stories, photos and linguistic expressions in the program, and also expert review of the same content. We will compare the unguided versus guided versions of iCBT to TAU to cover a continuum of least resource intensive/greater self-care options to the more standard highly dependent of professional resources options.

#### Intervention description {11a}

The guided and unguided iCBT interventions delivered through the SilverCloud platform can be accessed through the web interface or by downloading a smartphone app. The program comprises 7 core modules and 9 supplemental modules (see Table 1 for a description) that implement evidence-based CBT strategies for cognitive restructuring, behavioral activation, and relaxation training. The program uses the same techniques as face-to-face CBT, but these are communicated to the user via written text, graphics, videos, and interactive tools. Sessions consist of texts, testimonials, audios, educational video clips, and interactive quizzes, exercises, and homework. The platform is available 24/7 and the users are recommended to complete one module per week. In the case of the guided version, supporters (described further below) welcome users to the platform upon signup and thereafter provide weekly asynchronous written feedback on the activity and tools that users have undertaken on the platform and provide personalized recommendations of content for the users. Supporters can also suggest that users revisit some sections, practice further certain exercises, or unlock access to the supplemental modules to address concomitant difficulties (such as sleep, self-esteem, the COVID pandemic, etc.) based on open-ended text provided by users, thus tailoring the program to each user’s needs. This support in the form of weekly written messages sent through the platform lasts for 8 weeks, but users can continue to access the program for 12 months. Supporters will be bachelor-level graduates of psychology or behavioral health programs who have been trained in the SilverCloud platform and in how to deliver feedback. The content of the unguided version is the same as the guided version, but there is no supporter to provide weekly feedback. Students in the TAU arm will receive whatever service their university normally provides. We will not control the content of TAU, which could be medication, psychotherapy, or referral to a community treatment provider, but we will document this content for use in post hoc analyses. During the COVID-19 pandemic, most university services have been modified to provide online one-on-one psychotherapy via videoconferencing platforms.

**Table 1:** SilverCloud iCBT modules, goals, and activities

Modules	Goals	Activities
**1. Getting Started**		
	• Learn about CBT • Learn about depression and anxiety • Introduce the Mood Monitor • Introduce the CBT Cycle • Learn how thoughts, emotions, physical sensations, and behaviors affect each other • Introduce personal stories • Connect with the present moment	• Mood Monitor • My CBT Cycles • Staying in the Present (Breathe) • Depression and anxiety Myths & Facts Quiz • Establishing goals
**2. Noticing Feelings**		
	• Learn about emotions and role in CBT Cycle • Recognize emotions that are difficult to cope with • Recognize physical sensations • Identify activities to target distressing physical sensations associated with depression and anxiety • Explore the impact of lifestyle choices on emotions and well-being • Relate to personal stories	• Emotions & Your Body Quiz • My CBT Cycles • Mapping Lifestyle Choices • Staying in the Present (Progressive Muscle Relaxation)
**4. Boosting Behavior**		
	• Learn about the link between mood and behaviors • Improve knowledge of common behavioral traps and how to beat them • Learn tips on how to get motivated during periods of low mood • Recognize the importance of pleasurable activities and achievements in boosting mood • Relate to personal stories	• Mood & Behavior Quiz • Your Backup & Support Network • My Motivational Tips • My Activities • Your Mood & Your Body • Activity Scheduling • Staying in The Present (Mindful Eating)
**5. Spotting Thoughts**		
	• Learn about the role of thoughts in depression and anxiety within the CBT Cycle • Recognize negative automatic thoughts • Understand and recognize thinking traps • Relate to personal stories	• Me & My Thoughts Quiz • My CBT Cycles • Staying in the Present (Watching Thoughts)
**6. Challenging Thoughts**		
	• Learn about hot thoughts and how to recognize • Learn to challenge negative thoughts • Learn how to overcome specific thinking traps • Recognize situations where it is necessary to use thoughts to cope	• Your Thinking Style Quiz • My Helpful Thoughts • My CBT Cycles • Staying in the Present (Watching Thoughts)
**7. Bringing it Together**	• Preparation for coming to the end of the program • Recognize the importance of social support in staying well • Identify warning signs • Planning for staying well • Set goals for the future	• Your Backup and Support Network • Staying Well Plan • Goals • Taking Stock • Staying in the Present (Sounds)
**Optional modules**		
	• Core beliefs	• Facing fears
	• Difficult moments (COVID-19 pandemic)	• Sleep difficulties
	• Managing grief	• My self-esteem and me
	• Managing anger	• Communication & relationships • Relaxation

#### Criteria for discontinuing or modifying allocated interventions {11b}

Participants in the follow-up surveys who report suicidal thoughts with some intent of acting on these thoughts will receive a closing statement at the end of their survey encouraging them to contact a mental health crisis number and will be told that they will be contacted by the clinical liaison at their university by the next workday. Similarly, participants in the guided iCBT who mention suicidal thoughts to their guide will be evaluated by their guide for intentionality and those with some intent of acting on these thoughts will be encouraged to contact the health crisis number, and the clinical liaison at their university will contact them. Those with a suicide attempt or unplanned hospitalization for any reason will be terminated from the intervention.

#### Strategies to assess and improve adherence to interventions {11c}

User engagement in Internet interventions can vary and can include user-related, content-related, and technology-related barriers and facilitators of engagement [43]. At the second week of inactivity, users will be sent a motivational email designed to improve empowerment and address treatment expectations and they will be offered a brief motivational video-conferencing session that will target user-related (e.g., expectations) and technology-related barriers.

#### Relevant concomitant care permitted or prohibited during the trial {11d}

Trial participants may receive any concomitant care. Moreover, students in all intervention arms on waitlist for university services may begin that treatment once they are off the waitlist. Receipt of concomitant treatment and type of treatment (e.g., psychotherapy, psychopharmacology) will be assessed at both follow-ups.

#### Provisions for post-trial care {30}

Access to SilverCloud will continue for 12 months after randomization for participants randomized to the two iCBT arms. Participants randomized to TAU will have the opportunity to access SilverCloud after 12 months.

### Outcomes {12}

The primary outcomes will be depression and anxiety symptom reduction and remission at 90 days and 12 months measured by the widely-used Patient Health Questionnaire-9 (PHQ-9) [44], Generalized Anxiety Disorder-7 Scale (GAD-7) [45], and the PHQ-Anxiety and Depression Scale (PHQ-ADS) [46]. The PHQ-9 is a self-report measure of depressive symptoms in the prior 2 weeks. Scores range from 0 to 27 with higher scores representing greater depressive symptomatology. The scale has both a sensitivity and specificity of 88% for identifying MDD [44]. The GAD-7 is a self-report measure of anxiety symptoms in the prior 2 weeks. Scores range from 0 to 21 with higher scores representing greater anxiety. Sensitivity and specificity for identifying GAD range from 70 to 92% [47]. The PHQ-ADS is a composite measure of the PHQ-9 and the GAD-7. Primary outcomes will be assessed both dimensionally and categorically. Consistent with standard practice, categorical thresholds for treatment response will be outcome scores less than 50% of baseline scores [48]. Remission will be defined as 0–4 on the PHQ-9 [49], 0–4 on the GAD-7 [45], and 0–9 on the PHQ-ADS [46].

Secondary outcomes also measured at 90 days and 12 months will be assessed only dimensionally (that is change in means/medians between groups). A secondary outcome will be impairment as measured by self-report questions based on the Army STARRS survey [50] of role impairment due to mental and physical health in four life areas in the prior 2 weeks. Scores in each area range from 0 to 10 with higher scores reflecting greater impairment. Additional secondary outcomes will include symptoms of comorbid disorders: panic disorder, social phobia, binge-purging disorders, PTSD, insomnia, suicidality, and frequency and quantity of alcohol and drug use. Panic disorder symptoms will be assessed with the Composite International Diagnostic Interview Screening Scales (CIDI-SC [51, 52]); social phobia with Diagnostic and Statistical Manual of Mental Disorders, 5^th^ Edition (DSM-5) diagnostic criteria [53]; binge/purge with questions from the Primary Care Evaluation of Mental Disorders (PRIME-MD) Patient Health Questionnaire (PHQ) [54]; PTSD with the six-item short-form PTSD Checklist for DSM-5 (PCL-5) screening scale [55]; and insomnia with a reduced version of the Insomnia Severity Index (ISI) [56]. Modified versions of the Columbia Suicidal Severity Rating Scale (C-SSRS) [57] and the Self-Injurious Thoughts and Behaviors Interview (SITBI) [58] will also be used to assess the degree of suicidal ideation and the number of plans and attempts. These scales have evidence of good concordance with blinded clinical diagnoses.

### Participant timeline {13}

**Fig. 1:**
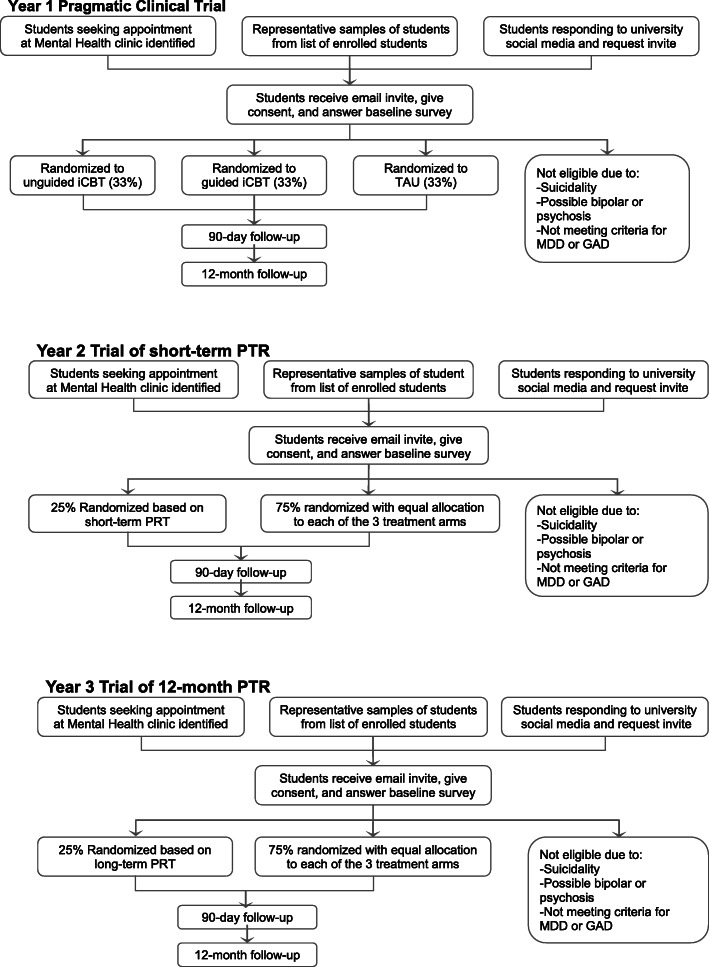
Participant timeline

**Fig. 2:**
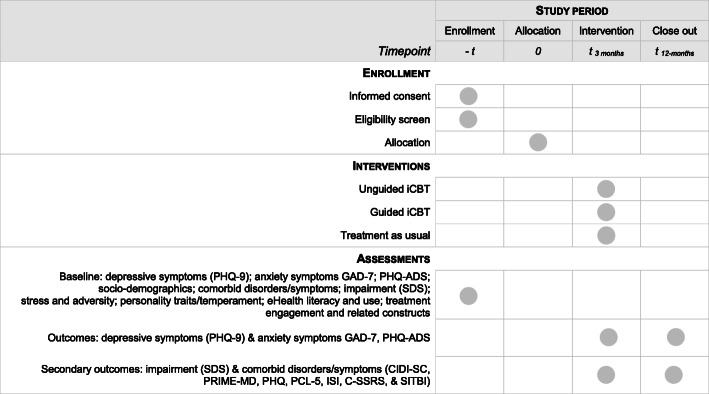
Schedule of enrollment, intervention, and assessments for pragmatic trials 1, 2, and 3

The participant timeline for each of the three trials is outlined in Fig. 1. Once students are identified through one of the three recruiting methods, they will be sent through email the study invitation, consent form, and baseline survey. Those who are not eligible to participate will be followed up by the clinical liaison at their university and provided the appropriate treatment. Those who are eligible will be randomized to one of the three treatment arms and will subsequently be asked to complete follow-up surveys at 90 days and 12 months after randomization. In the second- and third-year trials, participant flow is similar except that 25% of eligible participants will be randomized according to the PTR generated from the first-year pragmatic trial short-term and long-term effects in years 2 and 3 respectively and the remaining 75% will be randomized with equal allocation into the three treatment arms. The schedule of enrollment, intervention, and assessments for the three trials is outlined in Fig. 2.

### Sample size {14}

#### Expected number of subjects

We expect to randomize at least 1500 participants in the first year trial and 1000 in each of the second and third year trials.

#### Expected effect size and power calculations

We powered the study to estimate heterogeneity of treatment effects as defined by the increase in the proportion of patients we would expect to remit from the primary outcomes based on optimized rather than randomized treatment of 5%. We considered this the minimum difference of interest. Although we will be interested both in continuous (i.e., differences in mean scores on symptom rating scales) and categorical (i.e., probabilities of treatment response and remission) outcomes, we evaluated statistical power for a categorical measure of episode remission because this is of greatest clinical importance and because larger samples are required for powerful detection of effects on categorical than continuous outcomes. Meta-analyses of previous iCBT trials for CMDs show that treatment response is approximately 50% and remission is approximately 30%, with rates roughly half these values for control conditions. Closed-form solutions show that samples with fewer than 150 patients per arm provide adequate statistical power (.8 power based on .05-level two-sided tests) to detect effect sizes this large even after making reasonable adjustments for loss to follow-up. However, estimating power to detect heterogeneity of treatment effects is more complex because closed-form solutions do not exist and simulation is needed to determine required sample sizes.

We carried out such a simulation by building a series of hypothetical population models that assumed that each patient was randomized either to iCBT or a control condition; that the aggregate remission rates after adjusting for loss to follow-up were 27.5% and 17.5% in the two treatment arms, respectively; and that a series of relatively complex nonlinear-interactive multivariate associations existed between 20 predictors and remission. The assumed distributions of predictors and interaction values among predictors were consistent with those found in previous studies. We varied these coefficient values in different models to maintain the same aggregate remission rates but to have the difference between remission rates based on randomized versus optimized treatment vary between 0 and 15%. In each model, the remission rate if intervention assignment was randomized was set at 10% (i.e., 27.5–17.5%), but the remission rate if intervention assignment was optimized (i.e., if each student who would have a better outcome if assigned to iCBT was, in fact, assigned to iCBT and all other students were assigned to the control condition, which in some cases could involve these students receiving treatment at the student clinic and having better outcomes than if they received iCBT) was varied between 0 and 15%. We used a state-of-the-art ensemble machine learning method detailed in the proposal to estimate heterogeneity of treatment response. We discovered that adequate statistical power (again defined as .8 power based on .05-level two-sided tests) to detect a difference between the two remission rates of 5%, which we considered the minimum difference of interest, would require a sample of at least 500 patients per treatment arm [59]. A difference of 5% would represent a 50% increase on the 10% aggregate base of the difference in the remission rate between intervention and control groups. Based on a review of previous precision treatment analyses of CMDs, a difference of this magnitude is plausible. Based on this result, we designed the study to have a sample size of 500 per treatment arm. As noted above in the discussion of the sample design, we will have 1500 eligible students to randomize across three arms in the first trial, making it possible for us to estimate a precision treatment model based on data obtained in this trial. However, as noted above in the section on study design, the 12-month follow-up data for these students will not be available until YR2 and it will be possible to use the results of the model for the trial 2 randomization for the first time in YR3. We will replicate the model with follow-up data for students randomized in YR2 and use the harmonized precision treatment model results obtained in YR3 for a final Trial in YR4.

### Recruitment {15}

There are three avenues of recruitment. The first is from the student mental health clinics. When students request an appointment for the health clinic, the person responsible for appointments and intake will identify potential participants, describe the study, answer questions, and provide to the study team the contact information for interested students. The second is from a list that university officials will provide of all enrolled undergraduate students. Each week a random sample of students will be sent an email saying that their university is concerned about the emotional wellbeing of their students, describing the study, and providing a personalized link to further information, the informed consent, and baseline survey. They will also be given contact information and can request a call if they want to ask questions or need further information. A third, and final, avenue of recruitment is through postings on the universities’ social media. Potential participants are told to send an email to request further information or obtain a link to the study.

## Assignment of interventions: allocation

### Sequence generation {16a}

Baseline survey results will be used to stratify randomization of eligible patients across study arms. This will be done automatically within the Qualtrics survey platform using the 3-way cross-classification of sex at birth, depression symptom severity and anxiety symptom severity.

### Concealment mechanism {16b}

The allocation is concealed in that the allocation sequence is automatically generated in the Qualtrics platform and simultaneous with assignment.

### Implementation {16c}

The allocation sequence will automatically be generated in the Qualtrics platform at the end of the baseline survey, and a screen will show participants to which group he or she has been assigned. Simultaneously, the Qualtrics platform will send an automatic message to the iCBT coordinator (in the case of participants assigned to iCBT) who will then send out instructions to access the iCBT intervention or in the case of participants assigned to TAU the Qualtrics platform will send out an automatic message to the university clinical liaison.

## Assignment of interventions: blinding

### Who will be blinded {17a}

Recruiters will be blinded at the time of recruitment to treatment assignment given that assignment will happen only after recruitment is completed. The data analysts will also be blinded to treatment assignment.

### Procedure for unblinding if needed {17b}

If active suicidality emerges, the participant will be flagged and reported to the principal investigator (PI) and university clinical liaison, the latter of which will call the student to assess risk and provide additional support.

## Data collection and management

### Plans for assessment and collection of outcomes {18a}

The baseline, follow-up, and needs assessment surveys will be self-report scales administered online through the Qualtrics platform. The survey program uses complex skip logic, developed for this study, based on participants’ responses to the questions to reduce respondent burden and increase completeness and accuracy of responses.

#### Baseline survey overview

The baseline survey will include questions of 6 types: (i) basic socio-demographics, (ii) CMDs, (iii) impairments associated with CMDs, (iv) current and past treatment of these disorders, (v) COVID-19 experience, (vi) eHealth literacy and use of digital interventions, and (vii) constructs found in previous research to be significantly prescriptive predictors of heterogeneity of CMD treatment response (see Table 2).

**Table 2:** Constructs^a^ found to be prescriptive predictors of CMD treatment response in two or more studies

**I. Socio-demographics**: Female, low parent education, marital status, distance of school from permanent residence, living arrangement while at school, employment while at school	
**II. Behavioral health history, triggers, and symptoms:** Symptoms of generalized anxiety disorder, panic disorder, separation anxiety disorder, social phobia, post-traumatic stress disorder, major depressive disorder, adjustment disorder with anxiety and/or depressed mood, insomnia, multimorbidity, history of suicidality, early age of onset, chronicity, high baseline severity, endogenous (depression), number prior/recurrent episodes, high psychomotor symptoms/low energy (depression), avoidance (experiential and behavioral), fear of negative evaluation, role impairment, personality disorder (Borderline Cluster C), psychotic-like symptoms	
**III. Stress and adversity:** Childhood maltreatment/trauma, parental bonding, high stress, interpersonal loss, poor physical functioning, poor cognitive functioning, poor social functioning, poor social support/relationships	
**IV. Personality traits/temperament:** Agreeableness, alexithymia, anxiety sensitivity, attentional control, attributional style, catastrophic cognitions, conscientiousness, cognitive distortions, emotional regulation, extraversion, hopelessness, low openness to experience, negative affect/neuroticism, non-secure attachment style, perceived control, stress reactivity, temperament	
**V. Treatment engagement and related constructs:** Adherence, health literacy, preferences for type of treatment, therapeutic alliance	
**VI.** Other: High cognitive dysfunction/low intelligence	

^a^See [60–64] for reviews of relevant studies

#### Needs assessment survey overview

The needs assessment surveys will include questions that overlap with, but are not identical to, those in the baseline survey: (i) basic socio-demographics; (ii) CMDs; (iii) impairments associated with CMDs; (iv) current and past treatment of mental disorders; students who have not been in treatment at all in the past 12 months will be asked if they ever had a need for treatment in the past 12 months and if they say yes, then they will be asked why they did not seek treatment (barriers) and will be offered enrollment in the trial; and (v) the prescriptive predictors of heterogeneity of CMD treatment response, the same as those administered in the baseline survey of the main trial (see Table 2).

#### Content of specific sections

##### (i) Socio-demographics

These will include sex, marital status, sexual orientation, distance of permanent residence from school, living arrangements, and employment, all of which are hypothesized based on previous research to be prescriptive predictors of CMD treatment response.

##### (ii) CMDs

MDD and GAD will be the primary foci of the intervention. The assessments of the primary outcome disorders will be the widely used PHQ-9 (depression) [44] and GAD-7 (anxiety) [45] scales. Clinical thresholds will be scores of 10+ on the PHQ-9 [65], 10+ on the GAD-7 [45], and 20+ on the recently-developed PHQ-ADS [46]. Eligibility for the trial will require students either to meet criteria for one or both of these disorders or of the PHQ-ADS. However, we will also evaluate other DSM-5 disorders: panic disorder, social phobia, binge eating disorder either with or without purging, PTSD, insomnia, bipolar disorder, adult ADHD, and frequency and quantity of alcohol and drug use (cannabis, cocaine, any other street drug, or a prescription drug either used without a prescription or used more than prescribed to get high, buzzed, or numbed out). Although a larger set of disorders is used in the face-to-face WMH surveys [66], the 9 in the WMH-ICS surveys include those associated with highest role impairment among college students [1]. Bipolar disorder will be assessed with the CIDI-SC [51, 52, 67] only for lifetime prevalence and, as noted above, will be exclusionary for the trial. We will also ask if they have ever been diagnosed with schizophrenia or psychosis as an exclusionary criterion. The other 8 disorders will be assessed either for lifetime (binge-purging disorders), 2-week (PHQ-9, GAD-7), 6-month (ADHD), or 30-day (the other disorders) prevalence. The baseline survey will also assess lifetime prevalence and course of GAD, MDD, and PTSD based on these being hypothesized prescriptive predictors of treatment response (Table 2). Panic disorder will be assessed with the CIDI-SC [51, 52], social phobia with DSM-5 diagnostic criteria [53], binge/purge with the PRIME-MD PHQ [54], PTSD with the six-item short-form PCL-5 screening scale [55], insomnia with a reduced version of the ISI [56], and ADHD with the Adult Self-Report Scale-V1.1 (ASRS-V1.1) [68] screener. Modified versions of the C-SSRS [57] and the SITBI [58] will also be used to assess suicidal ideation, plans, and attempts. As noted above, suicidality with intent (i.e., a current suicide plan with intent to act on the plan) will be exclusionary for the trial.

##### (iii) Impairments due to CMDs

Impairments due to CMDs will be assessed with the SDS [50].

##### (iv) Current and past treatment

Treatment of CMDs will be assessed with the treatment section of the World Health Organization (WHO) Composite International Diagnostic Interview (CIDI) [69].

##### (v) COVID-19 experience

A cross-national team of researchers convened by WHO developed a COVID-19 question battery to assess the experiences of university students around the world with COVID. Questions include having ever been diagnosed with COVID-19, knowing someone who died of COVID-19 and their relationship with the student, and stress the pandemic has caused in different areas of their life.

##### (vi) eHealth literacy and use of digital interventions

This will be assessed with the eHEALS: The eHealth Literacy Scale [70] as well as additional items developed for this study to measure reasons for using digital mental health interventions and preferred features of these interventions.

##### (vii) Prescriptive predictors

Our prescriptive predictor battery was developed by carrying out a systematic literature review of prescriptive predictors of heterogeneity of CMD treatment response with respect to CBT in randomized controlled trials, uncontrolled treatment trials, and prospective observational studies of pre-treatment predictors of treatment response. Replicated significant associations were found for more than two dozen baseline patient self-reported constructs. Space restrictions preclude us from reviewing this voluminous literature, but key constructs are summarized in Table 2 along with references to key review papers. Based on these results, we carried out a second systematic review of best available short-form measures of each construct. We worked with psychometricians in secondary analyses to create new short-form versions of existing scales when none existed [51, 55, 67, 68, 71–73]. It is noteworthy that no prior trial included more than a few of these constructs, making it impossible to know if any of them is significantly independent of the others. Ours will be the first study to include a comprehensive baseline assessment of these constructs.

#### Additional measures used in the treatment trial follow-ups

The 90-day and 12-month follow-ups will focus on both process and outcome measures.

##### Process measures

Process measures will include treatment initiation and type (given that students in all arms will be free to obtain treatment other than our iCBT) using questions from the CIDI [69], satisfaction with treatment based on questions adapted from the Client Satisfaction with Treatment Questionnaire-8 (CSQ-8) [74] and additional items developed for this study, and measures extracted from the *SilverCloud* logs for patients randomized to iCBT to determine the number of logins, time spent overall and per session, and number of modules entered.

##### Outcome measures

These were described above.

### Plans to promote participant retention and complete follow-up {18b}

Participants will be given a gift card worth $15 for completing the 90-day follow-up and another gift card for the same amount for completing the 12-month follow-up. Email reminders will be sent 2 and 4 days after the initial invitation email and we will then make phone calls over the next week in an effort to encourage students to complete the follow-up survey.

### Data management {19}

The Instituto Nacional de Psiquiatría Ramón de la Fuente Muñiz [National Institute of Psychiatry Ramón de la Fuente Muñiz] (INPRFM) study team will maintain a master dataset for all participants who were referred to the project for recruitment along with a record of whether they withdrew before or after completing the baseline survey or after initiating the intervention, were considered ineligible for participation, or were terminated from the study or completed the study. Protection of student privacy with respect to survey data will be achieved by assigning each participant a study identification number and then creating a separate de-identified file for sharing with the Harvard study team and for archiving. All data analyses will be carried out with the de-identified data file. In addition, data cleaning programs will be run to look for implausible or inconsistent answers.

### Confidentiality {27}

Access to personal information of participants will be restricted to relevant INPRFM study staff. Only when there is serious risk of suicidality will information be reported to the university clinical liaison.

### Plans for collection, laboratory evaluation, and storage of biological specimens for genetic or molecular analysis in this trial/future use {33}

Not applicable as no biological specimens were collected as part of this trial.

## Statistical methods

### Statistical methods for primary and secondary outcomes {20a}

#### Analysis of aggregate intervention effects

Aggregate differences in outcomes across arms will be estimated using an intent-to-treat (ITT) [75] analysis approach as well as using a complier average causal effect (CACE) [76] analysis approach. ITT estimates the impact of intervention among all students offered the intervention whether or not they participated. CACE, in comparison, estimates the impact of intervention only on the students who participated in iCBT. To estimate CACE, we will use two-stage least squares (2SLS) [77] and control function procedures [78]. To test the validity of the instrumental variable (IV) assumption in 2SLQ, we will assess balance in the covariate distributions across groups generated by the IV. We will also conduct sensitivity analyses to detect violations of the “independence to unmeasured confounders” assumption, the “exclusion restriction” assumption, and the “monotonicity” assumption as described by Baiocchi et al. [79]. Inverse probability weights (IPW) will be used in all these analyses to deal with loss to follow-up [80].

For each of the two outcome assessments (i.e., 90-day and 12-month), probability of a student being in the study at time *t* will be calculated conditional on information collected as of time *t − 1*. Flexible, nonparametric estimation methods will be used for confounder control [81]. The IPW as of time *t* will be based on the product of these conditional probabilities up through *t*. The treatment-specific mean outcome will be estimated using these weights for the individuals whose outcomes were observed at *t*. Under a coarsening at random assumption, this estimator converges with increasing sample size to the treatment-arm-specific mean outcome that would have been observed had all individuals remained in the study up through *t* [82]. This assumption is closely related to the strong ignorability assumption commonly used in observational studies [83]. Generalized linear models (GLMs) will be used to estimate ITT effects on continuous outcomes, making use of standard visual diagnostics to choose appropriate link functions and error structures [84].

We will report adjusted mean differences (AMDs) with design-adjusted 95% CIs (confidence intervals) and use Poisson regression with robust variance estimation for binary outcomes and report adjusted prevalence ratios (aPRs) with 95% CIs. We will calculate numbers needed to treat (NNTs). When data are missing, we will provide thorough summaries of reasons for missingness and proportions of missing data, test for differences in the characteristics of patients with and without missing data, and describe these and the implications of missing data for interpretation when we report results. As our IPW approach to missing outcomes data will be based on a missing-at-random (MAR) assumption, sensitivity analysis will be carried out based on the weaker missing-not-at-random (MNAR) assumption using pattern mixture modeling [85] in a generalized mixed model framework [86]. The predictors of success in obtaining outcome data at time *t* will include information obtained in prior waves of data collection along with data on intensity of efforts needed to obtain outcome data in a discrete-time survival framework (i.e., in response to the 1^st^, 2^nd^, or 3^rd^ telephone attempts). A range of assumptions about the distribution of the missing data will be made in this approach to investigate sensitivity of results [87]. We will record and report distributions and correlates of dropout and account for all patients in reports.

#### Analysis of heterogeneity of treatment intervention effects

Precision treatment models will be built as in similar studies [88–91]. The models are based on a special case of the super learner (SL) algorithm [92], an ensemble machine learning approach that uses cross-validation (CV) to select a weighted combination of predicted outcome scores across a collection of candidate algorithms that yields an optimal weighted combination according to a pre-specified criterion that performs at least as well as the best component algorithm. The candidate algorithms in SL can either be parametric or flexible machine learning algorithms, making SL less prone to model misspecification than traditional parametric approaches. The guarantee that SL performs at least as well as the best candidate algorithm allows a rich library of parametric and flexible candidate algorithms to be included. In comparison, the conventional approach is to estimate a model with main effects and interactions between prescriptive predictors and dummy variables for treatment indicators [93]. Predicted values based on this model are then used to estimate expected individual-level treatment outcome conditional on prescriptive predictors for each patient in each treatment condition. An estimate of the optimal treatment strategy for patient *p* is then obtained by comparing predicted values of the outcome across all treatment arms. However, the accuracy of this approach requires correct specification of both the (possibly nonlinear) main effects and the (possibly complex nonlinear and higher-order) interaction terms. SL has two advantages over this conventional approach [94]. First, it requires only correct specification of the interactions, not the main effects, as it directly estimates contrasts. Second, SL uses flexible component machine learning algorithms maximizing chances of capturing complex nonlinear and higher-order interactions [92, 95].

The value of guided and unguided iCBT at the individual level will be quantified using an approach roughly equivalent to calculation of NNT in aggregate analyses based on a cross-validated targeted minimum loss-based estimator (CV-TMLE) [96] of the attained improvement of the mean outcome under a treatment selection scheme that always selects the treatment option with the best predicted outcome compared to the mean outcome under balanced randomization, i.e., when *1/3* of students are randomly assigned either guided iCBT, unguided iCBT, or to TAU (which in many cases will result in the student not receiving any treatment). The difference score, sometimes referred to in linear interaction models as the *personalized advantage index* (PAI) [93], quantifies the effect of using a precision treatment assignment rule to determine which patients should receive which treatments. The CV-TMLE estimate of the PAI has minimal bias because it uses CV to separate the estimation of the optimal treatment strategy from the assessment of the estimated strategy’s performance and also by allowing for the incorporation of flexible estimation approaches for the regressions and conditional probabilities needed to define the attained improvement.

### Interim analyses {21b}

Interim analyses will be conducted only to assess adequate randomization (i.e., equal numbers of participants by sex and level of anxiety and depression in each of the three treatment arms) and patterns and predictors of attrition to inform and improve retention strategies. Ongoing data monitoring will also be conducted if requested by the Data Safety Monitoring Board (DSMB).

### Methods for additional analyses (e.g., subgroup analyses) {20b}

As noted above, we will carry out subgroup analyses that evaluate the significance of HTE and attempt to develop an individual PTR to make treatment assignments under balanced allocation that optimize the aggregate remission rate.

### Methods in analysis to handle protocol non-adherence and any statistical methods to handle missing data {20c}

Efforts will be made to minimize loss to follow-up by using contact information obtained at baseline to attempt to contact and encourage non-respondents to complete the follow-up assessment. Mixed mode methods will be used both in contact attempts and to obtain outcome information. We will also attempt to obtain marker information in cases where these participants are unwilling to complete the full follow-up assessment. Reporting of follow-up results will be based on a CONSORT (CONsolidated Standards Of Reporting Trials) checklist and flow diagram [97]. As noted above, we will use IPW to adjust for loss to follow-up [80]. The IPW as of time *t* will be based on the product of these conditional probabilities up through *t*. The treatment-specific mean outcome will be estimated using these weights for the individuals whose outcomes were observed at *t*. Under a coarsening at random assumption, this estimator converges with increasing sample size to the treatment-arm-specific mean outcome that would have been observed had all individuals remained in the study up through *t* [82]. This assumption is closely related to the strong ignorability assumption commonly used in observational studies [83].

### Plans to give access to the full protocol, participant-level data, and statistical code {31c}

Data will become publicly available 6 months after the trials are concluded. First, we will document and make available all imputations, weights, and constructed variables used in our analyses. Second, we will host project webinars to present an overview of the data and answer questions. The webinars will be widely advertised in listservs, including any such sites recommended by NIMH, as well as to National Institute of Health (NIH) training programs. An English version of the webinars will be hosted by the US collaborators. A Spanish version will be hosted by the Mexican collaborators. Third, we will set up and man a project Q&A (question and answer) website in which public users can ask questions and get answers about issues involved in working with the data. Again, the site will be both in English and Spanish. Fourth, we will gather up written versions of all questions asked by public users along with our written response (including any documentation or computer files sent along with these answers) and post these on the website in both English and Spanish as reference documents for future public users.

## Oversight and monitoring

### Composition of the coordinating center and trial steering committee {5d}

The co-PIs who have overall responsibility for coordinating the study will meet periodically with the expert consultants (selected for their experience and research trajectory with clinical trials in general and online interventions in particular), data management staff, the SilverCloud team, the implementation staff, and university liaisons as needed. As described below, there will also be a DSMB.

### Composition of the data monitoring committee, its role, and reporting structure {21a}

The Co-PIs will have overall responsibility for monitoring the integrity of study data and participant safety. In addition, an independent monitoring committee, the DSMB was established to provide oversight for the safe conduct of the study. The DSMB is comprised of four individuals entirely independent of the trial team and with expertise in clinical management of depression, electronic interventions, biostatistics, trial design, ethics, bilingual English-Spanish, and understanding of Latino culture. They will meet quarterly. The DSMB’s main responsibilities include reviewing protocols, consent procedures, and safety plans prior to study initiation, amendments thereafter, and monitoring study progress in terms of recruitment and retention of participants, adverse events, reasons for participant withdrawal, participant privacy, and protocol violations or deviations and will be guided by the NIMH policy governing independent DSMBs [98]. The DSMB will have access to all safety and data quality information collected and will have the authority to stop the study if it raises unacceptable risks to participants. The clinical psychologist supervisor will also oversee the participant safety protocols, the supporter performance, and timely follow-up to any adverse events or serious adverse events (SAEs) over the course of the study, as well as reporting of such events to the Co-PI Benjet and the DSMB.

### Adverse event reporting and harms {22}

Regardless of treatment arm, we will monitor the occurrence of two specific SAEs: suicide attempt and unplanned hospitalization from any cause. These will be recorded systematically for all study participants at each outcome assessment (90 days and 12 months). In addition, these SAEs (and any other unexpected adverse event) that are brought to the awareness of the trial team during the trial will be similarly reported and recorded but will not be included in the trial evaluation. Additionally, SAEs may be detected in the guided iCBT by the supporters, which will be reported immediately to the Clinical Supervisor and PI Benjet. An initial report of SAEs will be sent to the DSMB within 24 h of the reporting. In all cases of detection of suicide attempts, the student will be immediately told (either by the supporter when detected in the intervention or by automatic message when detected in the survey) that a representative of the student mental health clinic will contact them by the next business day and that they can call the mental health crisis line (which we will provide) prior to that time if they are at imminent risk. All SAEs that are considered study-related will be reported to the NIMH Program Officer within 10 business days and the student will be terminated from the intervention. Students who report suicidal thoughts and behaviors in the baseline survey (used to determine study eligibility) will be given mental health crisis line numbers, flagged, and the university clinical liaison notified to contact that student within 24 h to assess risk and provide help.

### Frequency and plans for auditing trial conduct {23}

Study activities, including those related to consent, randomization, data collection, and intervention, will be monitored on an ongoing basis by the study DSMB. As mentioned above, the DSMB will meet quarterly or more frequently as needed to monitor trial progress and participant safety. As mentioned previously, the DSMB members are independent from investigators and the sponsor.

### Plans for communicating important protocol amendments to relevant parties (e.g., trial participants, ethical committees) {25}

Changes to study protocol will be communicated to the DSMB during quarterly meetings, or through email if required between meetings and as needed to clinical and university partners and participants.

## Dissemination plans {31a}

Study results will be disseminated through webinars and written documents to university administrators and students throughout Latin America, at the participating universities as well as through a consortium of Schools of Psychology in Latin American Universities. We will also prepare scientific reports of the study results for publication in high impact journals.

We will not use any professional writers. At least one individual from each participating university and the central organizing team will be eligible to be an author on future publications if they contribute to conception, design, analysis and/or interpretation of data, and initial drafting or reviewing of drafts.

## Discussion

Both guided and unguided iCBT are promising tools to reduce the personal and societal burdens of mental disorders in general and in the university population of LMICs in particular. The interventions may increase the scalability, affordability, and convenience of treatment delivery. Moreover, the interventions expand the settings to which they can be applied, are flexible, and can use a non-professional workforce [99]. While less is known about relative acceptability of Internet interventions and more traditional treatment delivery methods, the COVID-19 pandemic has been a game changer and made Internet interventions not only more acceptable, but necessary and is likely to remain a cost-effective option once the pandemic is over [100, 101]. This research will provide valuable information to university administrators and government policy makers in the participating LMICs to document the burden of CMDs among university students, the extent of unmet need for treatment of these disorders, the effectiveness of inexpensive-scalable iCBT interventions to treat these disorders, and for whom iCBT is most effective; thus, the more resource-intensive interventions can be saved for those who would not benefit from lesser resource-demanding options. Additionally, this will permit us to build capacity to disseminate the methods used for screening, targeting, and intervention implementation so that they can be used in colleges and universities in LMICs throughout Latin America. Such an effort could have enormous public health implications for the region and serve as a model of evidence-based intervention planning and implementation for other parts of the world.

## Trial status

IRB Approval of Protocol Version 1.0; 6/3/2020. Recruitment began 3/1/2021. Recruitment is tentatively scheduled to be completed 5/30/2024.

## Abbreviations

2SLS: Two-stage least squares
ADHD: Attention deficit hyperactivity disorder
AMDs: Adjusted mean differences
aPRs: Adjusted prevalence ratios
ASRS-V1.1: Adult Self-Report Scale-V1.1
CACE: Complier average causal effect
CBT: Cognitive behavioral therapy
CIDI: Composite International Diagnostic Interview
CIDI-SC: Composite International Diagnostic Interview Screening Scales
CIs: Confidence intervals
CMD: Common mental disorder
CONSORT: CONsolidated Standards Of Reporting Trials
COVID-19: Coronavirus Disease 2019
CSQ-8: Client Satisfaction with Treatment Questionnaire-8
C-SSRS: Columbia Suicidal Severity Rating Scale
CV: Cross-validation
CV-TMLE: Cross-validated targeted minimum loss-based estimator
DSM-5: Diagnostic and Statistical Manual of Mental Disorders, 5th Edition
DSMB: Data Safety Monitoring Board
GAD: Generalized anxiety disorder
GAD-7: Generalized Anxiety Disorder-7 Scale
GLMs: Generalized linear models
HTE: Heterogeneity of treatment effects
iCBT: Internet-delivered cognitive behavioral therapy
ICS: International College Student survey
INPRFM: Instituto Nacional de Psiquiatría Ramón de la Fuente Muñiz [National Institute of Psychiatry Ramón de la Fuente Muñiz]
IPW: Inverse probability weights
IRB: Internal review board
ISI: Insomnia Severity Index
ITT: Intent-to-treat
IV: Instrumental variable
LMIC: Low-middle-income country
MAR: Missing-at-random
MDD: Major depressive disorder
MNAR: Missing-not-at-random
NIH: National Institute of Health
NIMH: National Institute of Mental Health
NNTs: Numbers needed to treat
PAI: Personalized advantage index
PCL-5: PTSD Checklist for DSM-5
PHQ-9: Patient Health Questionnaire-9
PHQ-ADS: Patient Health Questionnaire-Anxiety and Depression Scale
PI: Principal investigator
PRIME-MD PHQ: Primary Care Evaluation of Mental Disorders Patient Health Questionnaire
PTR: Precision treatment rule
PTSD: Post traumatic stress disorder
Q&A: Question and answer
SAE: Serious adverse event
SDS: Sheehan Disability Scales
SITBI: Self-Injurious Thoughts and Behaviors Interview
SL: Super learner
TAU: Treatment as usual
WMH: World Mental Health
WHO: World Health Organization
