# Correction: Study protocol for pragmatic trials of Internet-delivered guided and unguided cognitive behavior therapy for treating depression and anxiety in university students of two Latin American countries: the Yo Puedo Sentirme Bien study

**DOI:** 10.1186/s13063-024-08176-9

**Published:** 2024-06-17

**Authors:** Corina Benjet, Ronald C. Kessler, Alan E. Kazdin, Pim Cuijpers, Yesica Albor, Nayib Carrasco Tapias, Carlos C. Contreras-Ibáñez, Ma Socorro Durán González, Sarah M. Gildea, Noé González, José Benjamín Guerrero López, Alex Luedtke, Maria Elena Medina-Mora, Jorge Palacios, Derek Richards, Alicia Salamanca-Sanabria, Nancy A. Sampson

**Affiliations:** 1 Center for Global Mental Health, National Institute of Psychiatry Ramón de La Fuente Muñiz, Mexico City, Mexico; 2 Department of Health Care Policy, Harvard Medical School, Boston, MA USA; 3 Yale University, New Haven, CT USA; 4 Department of Clinical, Neuro and Developmental Psychology, Amsterdam Public Health Research Institute, Vrije Universiteit Amsterdam, Amsterdam, Netherlands; 5 Center for Global Mental Health, National Institute of Psychiatry Ramón de La Fuente Muñiz and School of Psychology, UNAM, Mexico City, Mexico; 6 Universidad Cooperativa de Colombia, Medellin, Colombia; 7 Universidad Autónoma Metropolitana, Mexico City, Mexico; 8 Universidad De La Salle Bajío, León, GT Mexico; 9 Departamento de Psiquiatría y Salud Mental, Universidad Nacional Autónoma de México, Mexico City, Mexico; 10 Department of Statistics, University of Washington, Seattle, WA USA; 11 SilverCloud Health, Dublin, Ireland; 12 E-Mental Health Group, School of Psychology, University of Dublin, Trinity College Dublin, Dublin, Ireland; 13 Campus for Research Excellence and Technological Enterprise, Future Health Technologies Programme, Singapore-ETH Centre, Singapore, Singapore

**Correction: Trials 23, 450 (2022)**

**https://doi.org/10.1186/s13063-022-06255-3**

Following the publication of the original article [1], a correction is needed in the description of the measurement of impairment on page 8, paragraph 2 and in the corresponding reference [50] as shown below.

Incorrect statement: “A secondary outcome will be impairment as measured by the Sheehan Disability Scales (SDS) [50], a self-report measure of impairment due to mental and physical health in four life areas in the prior 2 weeks.

Incorrect reference: “50. Sheehan DV, Mancini M, Wang J, Berggren L, Cao H, Dueñas HJ, et al. Assessment of functional outcomes by Sheehan Disability Scale in patients with major depressive disorder treated with duloxetine versus selective serotonin reuptake inhibitors. Hum Psychopharmacol. 2016;31(1):53–63. https://doi.org/10.1002/hup.2500 .

Correct statement: “A secondary outcome will be impairment as measured by self-report questions based on the Army STARRS survey [50] of role impairment due to mental and physical health in four life areas in the prior 2 weeks.”

Correct reference: “50. Ursano RJ, Colpe LJ, Heeringa SG, Kessler RC, Schoenbaum M, Stein MB; Army STARRS collaborators. The Army study to assess risk and resilience in servicemembers (Army STARRS). Psychiatry. 2014;77(2):107–19. https://doi.org/10.1521/psyc.2014.77.2.107 ”.

The original article has been corrected.

## References

[1] Benjet C (2022). Study protocol for pragmatic trials of Internet-delivered guided and unguided cognitive behavior therapy for treating depression and anxiety in university students of two Latin American countries: the Yo Puedo Sentirme Bien study. Trials.

